# Long term follow-up of women treated for screen detected atypical ductal hyperplasia or lobular neoplasia in a large UK screening centre

**DOI:** 10.1038/s44276-024-00113-2

**Published:** 2024-12-18

**Authors:** Nicole L. Brown, Susan Pritchard, Elaine F. Harkness, Yit Lim, Ashu Gandhi, Dafydd Gareth Evans, Anthony Howell, Sacha J. Howell

**Affiliations:** 1 Manchester University NHS Foundation Trust, Wythenshawe Hospital, Southmoor Road, Manchester, UK; 2 Division of Informatics, Imaging and Data Sciences, The University of Manchester, Manchester, UK; 3 Division of Cancer Sciences, The University of Manchester, Manchester, UK; 4 Division of Evolution, Infection and Genomic Sciences, The University of Manchester, Manchester, UK

## Abstract

**Background:**

Atypical ductal hyperplasia (ADH) and lobular neoplasia (LN) increase subsequent breast cancer (BC) risk. However, optimal surveillance and risk reduction regimes remain uncertain. We report management and outcomes of women with ADH and LN to provide data on potential screening/prevention strategies.

**Methods:**

Women diagnosed with screen detected ADH and/or LN between 2010-2018 at our institution were identified and demographic data, MDT decisions and BC diagnoses extracted from electronic patient records in 2019 and 2023.

**Results:**

Of 107 women, 74 were discharged to the NHS Breast Screening Programme and 33 were offered enhanced screening (ES). The proportion offered ES increased significantly over time (*p* = 0.037). 15/105 (14.3%) developed BC (median follow-up 117 months), 9 screen-detected and 6 symptomatic, with 3 interval cancers diagnosed 12–25 months following their last screen. 3/15 were lymph node positive and 13/14 invasive cancers were estrogen receptor (ER) positive. BC incidence rate was 1499.6/100,000 women/year (SIR = 4.7), lower in the first 5 years of follow-up compared with post 5 years.

**Conclusions:**

In women with ADH/LN most BCs occur beyond 5 years. ES regimens should therefore extend to at least 10 years and be at least biennial. Preventative therapy should be considered given the high BC SIR and ER positivity of subsequent tumours.

## Introduction

The UK National Health Service Breast Screening Service (NHS BSP) routinely invites all women aged 50 to commence 3 yearly mammographic screening to age 70, with the option to self-refer for three yearly screening thereafter. Since the inception of the NHSBSP the incidence of preinvasive breast cancer and benign breast disease (BBD) have risen [1]. BBD represents a spectrum of histopathological abnormalities lying in between normal breast tissue and preinvasive/invasive malignancy. In the NHS BSP the spectrum of breast histopathology is classified from B1 (normal) to B5 (malignant) with B3 denoting lesions of uncertain malignant potential [2]. Lesions are classified as B3 either due to their risk of upgrade to malignancy following further sampling of the same site, the associated increased risk of future bilateral BC development, or both. Two lesions fall into this latter category - atypical intraductal epithelial proliferation (AIDEP) and lobular neoplasia (LN) [3].

AIDEP is a term used to describe core biopsy (CB) or vacuum-assisted biopsy (VAB) samples containing ductal epithelial proliferation that, on further larger excision biopsies, could be considered atypical ductal hyperplasia (ADH) [3]. ADH is a lesion that resembles, but falls short of, low grade ductal carcinoma in situ (DCIS). This is either because the abnormal features fail to warrant a DCIS diagnosis, or the lesion is less than 2 mm in size/occurs in fewer than 2 duct spaces. NHS BSP pathology guidance suggests 4 g of tissue is required to accurately make a diagnosis of AIDEP [2]. LN refers to dis-cohesive epithelial cell proliferation within the lobules of the breast. It is a term typically used to describe biopsy samples which are not large enough to differentiate between atypical lobular hyperplasia (ALH) and classical lobular carcinoma in situ (LCIS), as these two lesions are cytologically identical and differ only by the degree of acinar distension [4]. ADH, ALH and LCIS are proven to be risk factors for the subsequent development of BC. Historically, the relative risk of BC development in women with ADH and ALH is 4 times that of the general population, with a cumulative incidence of 1–2% per year [5–7]. LCIS represents a higher risk lesion, with reported relative risks, of 7–10 and an annual incidence rate of 2–3% [7–9].

Guidance on the management of BBD centres on two key factors; the chance of upgrade to preinvasive or invasive breast cancer with further excision and the longer-term risk of breast cancer development and hence requirement for and frequency of ongoing surveillance. In the 2018 NHS Breast Screening multidisciplinary working group Vacuum Assisted Excision (VAE) following diagnosis of B3 lesions was recommended, with the exceptions of those difficult to diagnose histologically or associated with papillary lesions, for which open excision was advised. Following excision, annual mammographic surveillance for 5 years was felt to be ‘prudent’ followed by ongoing 3 yearly screening in the NHSBSP. However, the group acknowledged that data were lacking to properly advise on intervals and duration of screening [3]. Subsequent data from the Sloane atypia prospective cohort in England, including 19,088 person years of follow up after atypia diagnosis in the NHSBSP, suggest the short-term risks of breast cancer development are lower than expected following the introduction of digital mammography [10]. The authors concluded that annual mammography after atypia diagnosis may not be warranted but that more evidence is required. European guidelines differ in that open excision is recommended for ADH following diagnostic core biopsy or VAB, whereas VAE was considered appropriate for the majority of LN cases [11]. Although surveillance was indicated for many, in particular with LN, neither type, frequency nor duration were discussed. Similarly, the American Society of Breast Surgeons recommends open excision for most cases of ADH but not for most ALH or LCIS [12]. The US National Comprehensive Cancer Network guidelines on breast cancer screening and diagnosis recommends annual tomosynthesis +/−MRI from the point of diagnosis of ADH or LN, if the residual lifetime breast cancer risk is ≥20% [13].

The lack of detailed follow-up and risk-reduction guidance for women with ADH, ALH and LCIS is at odds with other high-risk groups, such as those with a strong family history of BC, for whom the National Institute for Health and Care Excellence (NICE) recommends tailored screening and prevention approaches [14]. Individualised risk prediction in women with ADH, ALH and LCIS is difficult. Even though several breast cancer risk prediction models do factor BBD into their calculations, including the Gail/BCRAT, Tyrer-Cuzick and Breast Cancer Surveillance Consortium (BCSC), their ability to correctly determine which patients with ADH and LN will develop BC are currently little better than chance alone [15–18]. In terms of preventive therapy NICE, European and NCCN guidance all suggest women with ADH or LN diagnosis should be offered treatment with breast cancer preventive medication given the positive results of clinical trials that included women with BBD as a risk factor [11, 14, 19–22].

The aim of this service review was to understand how women diagnosed with screen-detected ADH, ALH and LCIS in our large BC screening and surgical tertiary referral centre in Manchester, UK were managed. Through analysis of the number of patients developing BC and the method of detection we hoped to provide information to help determine future screening and prevention strategies.

## Methods

### Patient sample

Women with screen detected AIDEP, ADH and LN, were identified through two reports run on the National Breast Screening System (NBSS); one for women who had undergone an excision biopsy (EB) for benign disease, and another for women who had a CB or VAB diagnosis of a B3 lesion between 2010 and 2018 at The Nightingale Centre, Wythenshawe Hospital, Manchester, UK. The histopathology reports of these patients were then reviewed to confirm atypia and subdivide by histological diagnosis.

The numbers of women attending for screening, being diagnosed with screen detected breast cancer and having involved axillary lymph nodes (ALN), were determined from the North West regional screening service annual reports. Interval cancers were identified through the North West interval cancer registry and EPR searches undertaken to obtain ALN status.

### Data collection

Demographic and imaging data were collected from case notes and electronic patient record (EPR) in 2019. Data extraction began from the date of the screening mammogram that resulted in a diagnosis of atypia and included biopsy type, date and result, BC risk factors, demographic factors including age and ethnicity, multi-disciplinary (MDT) decision regarding mammography and chemoprevention and subsequent BC diagnoses.

BC risk factors of interest were family history, age of menarche, age at birth of first child, menopausal status, age of menopause, and use of previous hormonal replacement therapy (HRT). Ethnicity was categorised into ‘White’, ‘Black – British’, ‘Black – African’, ‘Black – Caribbean’, ‘Asian - Pakistani’, ‘Asian - Indian’, ‘Asian - Chinese’, ‘Mixed’ and ‘Not documented’. Overall histological diagnoses were grouped into the categories ‘ADH’, ‘AIDEP’, ‘LN’, ‘ADH and LN’, ‘AIDEP and LN’, and ‘atypia – other’. Not all patients had a VAE or EB, and AIDEP remained the most conclusive diagnosis. Due to the variability in the reports that separated ALH from LCIS, these were grouped together as LN. The American College of Radiologists BI-RADS 5th edition [23] was used to categorise breast density, which was reported from the contralateral breast at the time of BBD diagnosis by a single experienced consultant radiologist at the Nightingale Centre.

In August 2023, a second search of the EPR was conducted to identify additional BC diagnoses and tumour characteristics, method of detection and the number of mammograms between diagnosis of atypia and diagnosis of BC collected. Cases were censored at the first BC diagnosis, death or 15/08/2023, whichever came first.

### Individualised BC risk calculations

10-year risk of BC was calculated for patients with all factors documented in the BCSC model (age, race/ethnicity, family history of BC in a first-degree relative, history of BBD (prior biopsy - unknown diagnosis, non-proliferative lesion, proliferative changes without atypia, proliferative changes with atypia, LCIS) and BI-RADS breast density). LCIS was only selected if the pathology report specifically stated this. If the report stated LN, ‘proliferative changes with atypia’ was used. As the BCSC model does not have the option of ‘Chinese’ for ethnicity, ‘Other/multiple races’ was used in this instance. Risk categories were according to NICE criteria Family History Guidelines [14], with a ten-year risk of <3% for those aged less than 50 and <5% at age 50 and above considered population-risk, 3–8 or 5–8% respectively considered ‘moderate’ risk and >8% at any age considered high risk.

### Statistical analysis

Chi-squared test was used to determine whether there was an association between year of diagnosis and enhanced screening (ES), and Fisher’s exact test was used to determine whether there was any association between risk categories (predicted 10-year risk of BC) and subsequent BC development. Statistical tests were carried out using SPSS version 25.0.0.1. Kaplan–Meier log rank analysis was used to compare risk variables with BC incidence.

Incidence rates of BC and 95% confidence intervals were calculated using the total number of follow-up years and the total number of patients diagnosed with BC within this period [24].

### Ethical approval

The project was approved by Manchester University NHS Foundation Trust as a service evaluation (reference number S257). Service evaluation in England is exempt from ethics committee review (Health Research Authority Guidance; www.HRA.NHS.UK).

## Results

NBSS searches identified 136 eligible patients with a B3 diagnosis at our institution. Twenty nine were subsequently excluded; 21 did not have ADH, LN or AIDEP (flat epithelial atypia (FEA; *n* = 14), papilloma with atypia (*n* = 3), atypia unspecified (*n* = 2) and 1 each for epithelial proliferation and atypical apocrine adenosis), 2 transferred to the private sector, 2 moved away from the area, 2 had no paper or electronic notes 1 presented symptomatically with ADH and 1 did not have a diagnosis of atypia. The remaining 107 patients were included in the final analysis. The median age at diagnostic screening mammogram was 53 years (range 47–74). Further demographic, diagnostic and risk factor data are presented in Table 1. BC risk factor documentation was variable, with only 40.0% having a full set of risk factors documented and 34.3% having no documented risk factors. Family history was the most frequently recorded risk factor (62.6% of patients).

**Table 1:** Histology, demographic and risk factor data.

	Number of patients
Histology (*N* = 107)	
ADH	44 (41.1%)
LN	46 (43.0%)
AIDEP	5 (4.7%)
ADH, LN	11 (10.3%)
AIDEP, LN	1 (0.9%)
Diagnostic pathway (*N* = 107)	
CB only	3 (2.8%)
CB → VAB/VAE	31 (29.0%)
CB → VAB → EB^a^	37 (34.6%)
CB → EB^b^	36 (33.6%)
Ethnicity (*N* = 107)	
White	76 (71.0%)
Asian (Pakistani, Indian, Chinese) or Mixed	6 (5.6%)
Unknown	25 (23.4%)
BIRADS Density (*N* = 107)	
A	8 (7.5%)
B	53 (49.5%)
C	36 (33.6%)
D	10 (9.4%)
Family History (*n* = 105^c^)	
None	49 (46.7%)
First-degree	8 (7.6%)
Second-degree	8 (7.6%)
Unknown	40 (38.1%)
Age of Menarche (*n* = 105^c^)	
11–13	30 (28.6%)
14–16	16 (15.2%)
Unknown	59 (56.2%)
Age of First Pregnancy (*n* = 105^c^)	
14–19	9 (8.6%)
20–24	16 (15.2%)
25–29	11 (10.5%)
30–34	9 (8.6%)
Nulliparous	10 (9.5%)
Unknown	50 (47.6%)
Menopausal status (*n* = 105^c^)	
Premenopausal	14 (13.3%)
Perimenopausal	7 (6.7%)
Postmenopausal	49 (46.7%)
Unknown	35 (33.3%)
Age of menopause (*n* = 49)	
42–45	5 (10.2%)
46–50	12 (24.5%)
51–55	7 (14.3%)
Unknown	15 (30.6%)
Previous Hormone Replacement Therapy (*n* = 49)	
Yes	18 (36.7%)
No	21 (42.9%)
Unknown	10 (20.4%)

*ADH* atypical ductal hyperplasia, *LN* lobular neoplasia, *AIDEP* Atypical IntraDuctal Epithelial Proliferation, *CB* Core biopsy, *VAE* vacuum assisted excision, *VAB* vacuum assisted biopsy, *EB* excision biopsy, *BIRADS* Breast Imaging and Reporting Data System.

^a^One without CB.

^b^2 with FNA prior to CB.

^c^Paper records were not available for 2 patients.

### MDT outcomes

Of the 107 patients, 74 were discharged back to NHS BSP 3 yearly mammography (including 4 over the age of 70 who would be expected to self-refer) [25]. The remaining 33 patients were offered ES; either one additional mammogram (*n* = 13) or annual screens for 5 years (*n* = 20), with one of these women also offered MRI within the first year and another offered extended annual screening to 10 years. Of the 13 cases in which one additional mammogram was offered, 4 were not performed for unknown reasons. Of those performed, the median time from initial biopsy to the additional mammogram was 13 months (range 11–36 months).

Of the 20 patients offered annual mammograms, the median number attended was 4. Only 5 patients had all 5 annual mammograms performed although 4 did not attend in 2020 likely reflecting suspension of breast screening during the Covid-19 pandemic. The 1 patient offered ES for 10 years has continued to have annual mammograms. 1 patient’s up to date notes could not be accessed, 2 patients developed BC within the 5-year screening period and 1 patient was not due her 5^th^ mammogram until after data collection ended. Notably, 1 patient was rediscussed at MDT after 2 years of annual mammograms and offered only one further annual screen and another was discharged back to the NHS BSP by her surgeon after 3 years of annual mammograms.

**Fig. 1:**
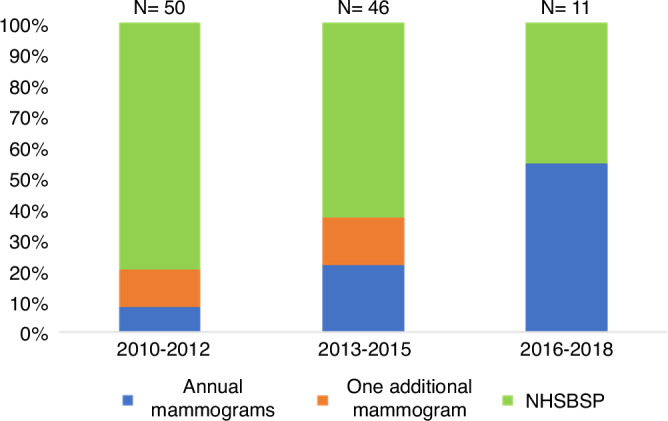
Proportion of patients offered enhanced screening by year of diagnosis. The proportion of women offered annual (blue), vs one additional (orange) vs 3 yearly NHSBSP (green) mammogaraphy in each of three study time periods.

The number of patients offered ES was associated with year of diagnosis, with ES increasing significantly over time (X^2^ = 6.459, *p* = 0.037; Fig. 1). Preventive therapy was only offered to 3 patients, tamoxifen (declined) for 1 patient and entry into the IBIS-II trial for 2 with the decisions unknown [20].

### BC risk prediction

52 patients had all risk factors documented for calculation of BCSC risk scores. The mean 10-year risk was 8.2% (95% CI 7.5–8.8%). Using these scores, 4/52 (7.7%) patients were considered population risk, 20/52 (38.5%) moderate risk and 28/52 (53.8%) high risk.

### BC diagnoses

Of the 105 patients whose medical records were accessible in 2023, 15 (14.3%) patients developed BC within the estimated median follow-up time of 117 months (range 11–162 months). Further information regarding tumour characteristics and previous atypia histology is shown in Table 2. The median time from B3 biopsy to BC diagnosis was 87 months (range 10–124 months), with a median of 2 intervening mammograms (range 0–7). The median age at diagnosis of BC was 60 years (range 55–79 years). Details regarding method of diagnosis, time to diagnosis and lymph node status for each screening plan can be found in Table 3. Two patients had BC detected by annual screening mammography (at the 4^th^ and 5^th^ screen), though the latter was 5.8 years after atypia diagnosis due to delays in annual mammograms. Two further patients allocated to receive annual mammograms presented symptomatically with BC, one before the first and one 20 months after the final annual mammogram.

**Table 2:** Tumour characteristics, method of diagnosis and previous atypia histology of BCs developed following atypia diagnosis.

	Number of BC cases (*N* = 15)
Invasive	14
Surrounding DCIS	8
No surrounding DCIS	6
Non-invasive	1
Site	
Ipsilateral	9
Contralateral	6
Invasive tumour type	
Ductal	12
Lobular	2
Grade	
1	5
2	7
3	2
T stage	
1	12
2	1
3	1
4	0
N stage	
0	9
1	3
2	0
3	0
Not assessed	2
ER status	
Positive	13
Negative	1
PR status	
Positive	12
Negative	2
HER2 status	
0	1
+1	9
+2 ISH negative	4
+2 ISH positive	0
+3	0
Atypia diagnosis	
ADH	8
LN	3
ADH + LN	3
AIDEP	1

*DCIS* Ductal Carcinoma In Situ, *ER* Estrogen Receptor, *PR* Progesterone Receptor, *HER2* Human Epidermal Growth Factor Receptor 2, *ISH* In Situ Hybridisation. Atypia abbreviations as Table 1.

**Table 3:** Method of BC Diagnosis and Lymph Node status by planned screening approach.

Screening plan	Number of cancers	Screen detected (months after atypia diagnosis)	Symptomatic (months after atypia diagnosis)	Diagnosed by NHSBSP after ES?	Symptomatic after ES?	Lymph node^b,c^
NHS BSP	9/74	5/9^c^ (38, 43, 105, 105, 105)	4/9 (72, 87, 115, 124)	–	–	1/9
1 additional screen	2/13	2/2 (33, 89)	0/2	2/2	0/0	1/2
Annual for 5 years^a^	4/20	2/4 (61, 70)	2/4 (10, 87)	0/2	1/2	1/4
Total	15/107	9/15	6/15	2/4	1/2	3/15

*NHSBSP* National Health Service Breast Screening Programme, *ES* enhanced screening.

^a^1 patient to 10 years and 1 had MRI for 1 year.

^b^All 3 cases of lymph node positive BC were screen-detected.

^c^One BC detected incidentally on mammogram performed due to symptoms in the other breast.

**Fig. 2:**
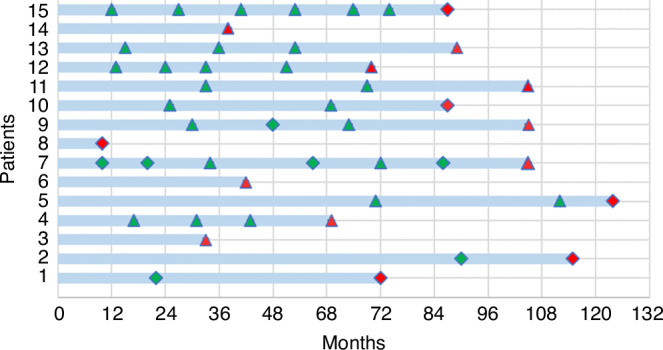
Time from atypia diagnosis to BC diagnosis with intervening mammograms. *Patient 7 – BC detected incidentally on mammograms performed due to symptoms in the contralateral breast. Green triangle: Screening mammogram – negative, Red triangle: Screening mammogram – positive, Green diamond: Symptomatic mammogram – negative, Red diamond: Symptomatic mammogram – positive.

Four patients had symptomatic BC in the cohort discharged to the NHS BSP. Two were interval cancers, diagnosed at 12 and 25 months after the last screening mammogram. The remaining 2 patients were diagnosed 72 and 115 months after atypia diagnosis but did not have any NHS BSP mammograms during this time. Three BCs were detected by the patient’s first NHS BSP mammogram following atypia diagnosis. Of these, 1 was lymph node positive. One cancer was diagnosed by the second NHS BSP mammogram, and 2 by the third. All three of these BCs were lymph node negative. The final BC was detected on mammograms performed due to symptoms in the contralateral breast, 18 months after the last mammogram. Details of both screening and symptomatic mammograms and time to BC diagnosis for each patient are shown in Fig. 2.

In our cohort 3/15 women (20.0%) had BC with involved axillary lymph nodes. In comparison, between 2021 and 2023 there were 123,021 screening mammography attendances in the North West of England NHSBSP of which 1,035 (0.84%) resulted in a diagnosis of screen detected breast cancer, 134 (12.95%) of which had involved axillary lymph nodes. During the same period a further 171 interval cancers were confirmed of which 58 (33.92%) had involved axillary lymph nodes and in total, therefore, there were 1206 breast cancers diagnosed in women participating in the NHSBSP of which 192 (15.92%) had involved axillary lymph nodes.

Using the total estimated follow-up years of 1000.25, the incidence rate for development of BC in this cohort was 1,499.63 women per 100,000 per year (95% CI 904–2487), a standard incidence ratio (SIR) of 4.73 (95% CI 4.50–4.97) (the population average in 2016–2018 for women aged 45–74 was 317.17 per 100,000 women per year) [26].

The rate of BC development in the first 5 years of follow-up was 773.32 per 100,000 women per year compared with 2277.43 per 100,000 women per year post 5 years (rate ratio 2.95 (95% CI 0.96–10.69, *p* = 0.058).

In the 52 patients with all risk factor data collected for BCSC risk algorithm completion, those considered low-moderate risk of BC were significantly more likely to develop BC than those considered high risk (6/24 low-moderate risk, 0/28 high risk; *p* = 0.007). Kaplan–Meier log rank analysis found no significant association between BC family history and subsequent risk of BC (X^2^ = 1.279; *p* = 0.28) although the number with a recorded family history was low (Table 1). Similarly, there was no significant difference in the risk of BC by type of BBD (ADH/AIDEP vs LN; X^2^ = 1.707; *p* = 0.191). However, a significant inverse association between mammographic density (BIRADS A/B vs C/D) and BC development was observed (X^2^ = 6.195; *p* = 0.013) although no correction for body mass index could be made as these data were not available.

## Discussion

We report the management and follow-up of patients diagnosed with ADH and LN at a large tertiary referral breast screening centre. We confirm that ADH and LN are high-risk lesions with a cumulative incidence of 14% and an SIR of 4.7 after a median follow-up of 117 months. These figures are comparable to prior studies reporting an annual incidence of between 1 and 3% and a SIR of between 4 and 10 for atypical hyperplasia (AH) and LCIS respectively. ^5–9^ It is also in keeping with a recent study which also included patients with ADH, ALH and LCIS, that found 9.9% developed BC over a follow-up time of 7 years [27].

Two recent, large retrospective cohort studies from the UK [Sloane] and US [BCSC] have shown that the incidence of BBD with atypia (ADH only in [BCSC]) has increased with the introduction of screening mammography and further again with the switch to full-field digital mammography (FFDM) [10, 28]. The Sloane study reported a fall in the incidence of breast cancer (preinvasive and invasive) within 3 years of an atypia diagnosis by fourfold between 2003-07 (24.3 invasive cancers per 1000 women; 95% CI 13.7–40.1) and 2013-18 (6.0; 95% CI 3.1–10.9) which led the authors to conclude that the current UK guidance to perform annual mammography for 5 years after BBD with atypia diagnosis may not be justified. Long term follow-up of the more recent cohort was not available and more research into the optimal screening strategy post diagnosis was suggested [10]. In the US study only subsequent invasive breast cancer was considered, in women diagnosed with ADH specifically. Cases with LCIS prior to cohort entry were excluded and those diagnosed during the study were censored in the analysis. ALH cases and any subsequent cancers arising in these women were neither excluded nor censored. This may have contributed to the relatively modest increase in incidence of invasive BC overall (Hazard Ratio 2.6; 95% CI 2.0–3.4). Interestingly the invasive cancer incidence did not differ significantly by time from ADH diagnosis (0–2 years HR 3.2 (95% CI 1.9–5.3), 2.01–4.99 years HR 2.4 (1.4–4.0) and 5–10 years HR 2.7 (1.7–4.3)) [28]. In a recently published update of the Mayo Clinic BBD cohort, in women diagnosed with all types of BBD by percutaneous biopsy but without subsequent excision in the majority (~80%), the cumulative incidence of invasive BC increased in a linear manner over time [29]. In contrast, our smaller cohort shows an increased risk of breast cancer in the second five years after excision of B3 lesions with atypia. This was also observed in a multicentre cohort of women diagnosed with B3 lesions in Switzerland, that did not undergo excision after VAB diagnosis, which demonstrated a ‘late peak’ in invasive breast cancer diagnosis after 7–8 years of follow up [30]. The differences in the patient populations, routes to diagnosis and subsequent management strategies highlights the need for ongoing long term data collection in this high-risk group.

Two thirds of patients in our cohort were not offered enhanced mammographic screening, though implementation of 5 years of annual mammograms did increase over time, likely reflective of changing attitudes and guidance [3]. MDT notes from the individual participants did not provide insight into the rationale for the differences in screening recommendations. Variability in approach to risk assessment and management of patients with AH/LCIS has previously been documented in US studies and was certainly evident in our istitution [31, 32]. Only 3 of 107 patients had evidence that they were offered risk reducing medication or clinical trials, suggesting this was not a priority for the breast MDT.

Of note, the median time from atypia to BC diagnosis was 87 months, with 11 of the 15 patients being diagnosed with BC greater than 5 years after a diagnosis of ADH or LN. These findings are similar to those in the aforementioned study of both ADH and LN, in which median time to diagnosis was 71 months [27], and a large UK observational study of patients with DCIS, in which the cumulative risk of BC increased significantly from 3 years following DCIS diagnosis and continued to diverge from population risk over time [33]. Most recently the Sloane atypia prospective cohort reported cumulative incidence rates of approximately 0.01%, 1.4% and 4.5% at one, three, and six years after atypia diagnosis, the authors suggesting that annual mammography for five years may not be beneficial [10]. The results presented here also call into question whether 5 years of annual screening is optimal and certainly highlights the ineffectiveness of one additional mammogram at 1–2 years. Instead, we suggest that more prolonged surveillance for at least 10 years, depending on fitness and suitability for ongoing screening, would be a more appropriate follow-up plan. Regarding the frequency of screening, the occurrence of interval cancers in those undergoing NHS BSP 3-yearly mammograms and the proportion with ALN involvement that is numerically greater than those in the 3 yearly NHSBSP in the same period, suggests more frequent mammograms may be required for patients with ADH and LN, however drawing firm conclusions on exact screening intervals from our small cohort is difficult. Given that our patients presented symptomatically 12–25 months after their last mammogram, either annual or biennial mammograms could be considered.

At present, a ‘one size fits all’ approach to follow-up is used for women diagnosed with ADH and LN, as BC risk prediction models have shown poor discrimination for women with atypia [15, 18], with several models either significantly over- or underestimating their risk of subsequent breast cancer [16, 17, 34]. This is thought, in part, to be due to conflicting data regarding the interaction of ADH, ALH and LCIS and other risk factors such as family history and breast density [35–37]. In our study, neither the type of BBD nor the presence of family history of BC were significantly associated with subsequent BC development. However, we cannot exclude potentially important differences in incidence and timing of invasive cancers after BBD subtypes due to the small numbers and histological heterogeneity in each cohort. Tailored follow up strategies based on BBD subtype could be considered with results from larger cohorts. The observed inverse association of BC risk and mammographic density was unexpected and may be confounded by our inability to correct for body mass index. Other studies have shown significant positive associations between density and risk in women with BBD with atypia [35, 36, 38]. Additional follow up of the Sloane atypia cohort will hopefully shed more light on this important subject. In the absence of a more refined risk prediction approach, we suggest all with ADH or LN be offered additional mammographic follow up.

Whilst we have demonstrated that risk of BC is substantially raised in patients with ADH and LN, reassuringly, the majority of these cancers were early stage, hormone receptor-positive, HER-2 and lymph node-negative and therefore of good prognosis. These characteristics are well documented in the literature, with previously reported figures of two thirds to three quarters being lymph node negative and 85% being hormone receptor positive [5, 8, 27]. The tendency for these tumours to be hormone receptor positive explains the effectiveness of chemoprevention in reducing the risk of subsequent BC in this population. For example, in women with a prior diagnosis of BBD undergoing preventive therapy in clinical trials, BC incidence was reduced by 86% with tamoxifen in NSABP P1 and by 69% with anastrozole in IBIS-2 [20, 21]. These data suggest that women with BBD should be offered preventive therapy with tamoxifen if premenopausal and anastrozole if postmenopausal and supported with management of side effects to facilitate adherence.

The main strength of this study is the relatively long-term follow up of women diagnosed with histologically confirmed ADH and LN in a specialist UK breast screening centre. As previously mentioned, the main limitation is the relatively small sample size, with only 15 patients being diagnosed with BC. There is also the potential for incomplete ascertainment as women could have moved away from the area without our knowledge. Thus, the true cumulative incidence of BC could have been higher as those with no follow up data were assumed not to have developed BC. We also found that a large proportion of patients had no BC risk factors documented, meaning that individual BC risk could only be calculated for half the cohort. Finally, as we used the NHS BSP database to identify patients, only those who had their ADH and LN detected by screening mammograms were included. Further evaluation of a larger cohort of patients presenting both through screening and symptomatically is required to provide clear follow-up guidance, ensuring that data is reflective of all patients.

## Conclusions

Women with ADH and LN have 4.7 times the risk of subsequent breast cancer development compared with the general population. Most breast cancers occur beyond five years and improved screening strategies, over those reported here, are required. Our data suggest optimal screening regimens for women with ADH or LN should extend to at least 10 years and be at a minimum of 2 yearly intervals. Women with ADH and LN should also be given the opportunity to take preventive therapy with tamoxifen or anastrozole.

## Data Availability

The datasets generated during and/or analysed during the current study are available from the corresponding author on reasonable request.
